# Isolation and characterization of novel *Fusobacterium nucleatum* bacteriophages

**DOI:** 10.3389/fmicb.2022.945315

**Published:** 2022-11-03

**Authors:** Yue Wang, Zhitong Liu, Qian Chen, Liqi Yi, Zihao Xu, Mufeng Cai, Jinhong Qin, Yan Zhang, Guanhuan Du, Jie Hong, Xiaokui Guo, Chang Liu

**Affiliations:** ^1^Department of Immunology and Microbiology, Shanghai Jiao Tong University School of Medicine, Shanghai, China; ^2^Research Institute of Intestinal Diseases, Shanghai Tenth People’s Hospital, Tongji University School of Medicine, Shanghai, China; ^3^School of Global Health, Chinese Center for Tropical Diseases Research, Shanghai Jiao Tong University School of Medicine, Shanghai, China; ^4^Department of Oral Mucosal Diseases, Shanghai Ninth People's Hospital, Shanghai Jiao Tong University School of Medicine, Shanghai, China; ^5^College of Stomatology, Shanghai Jiao Tong University, National Center for Stomatology, National Clinical Research Center for Oral Diseases, Shanghai Key Laboratory of Stomatology, Shanghai, China; ^6^State Key Laboratory for Oncogenes and Related Genes, Key Laboratory of Gastroenterology and Hepatology, Ministry of Health, Division of Gastroenterology and Hepatology, Shanghai Cancer Institute, Shanghai Institute of Digestive Disease, Renji Hospital, Shanghai Jiao Tong University School of Medicine, Shanghai, China; ^7^Key Laboratory of Parasite and Vector Biology, Ministry of Health, Shanghai, China

**Keywords:** *Fusobacterium nucleatum*, anaerobe, bacteriophage, phage therapy, morphology, genome

## Abstract

*Fusobacterium nucleatum* is a strictly anaerobic, Gram-negative bacterial species that is a member of the commensal flora in the oral cavity and gut. Recent studies suggested that the increase of abundance is associated with the development of various diseases, among which colorectal cancer is of the biggest concerns. Phage therapy is regarded as a potential approach to control the number of *F. nucleatum*, which may contribute to the prevention and treatment of related diseases. In this study, we isolated five isolates of bacteriophage targeting *F. nucleatum*. The morphological, biological, genomic and functional characteristics of five bacteriophages were investigated. Transmission electron microscopy revealed that JD-Fnp1 ~ JD-Fnp5 are all myoviruses. The size of the JD-Fnp1 ~ JD-Fnp5 genomes was 180,066 bp (JD-Fnp1), 41,329 bp (JD-Fnp2), 38,962 bp (JD-Fnp3), 180,231 bp (JD-Fnp4), and 41,353 bp (JD-Fnp5) respectively. The biological features including pH and heat stability, host range, growth characteristics of JD-Fnp1 ~ JD-Fnp5 displayed different patterns. Among them, JD-Fnp4 is considered to have the greatest clinical application value. The identification and characterization of JD-Fnp1 ~ JD-Fnp5 provides a basis for subsequent therapeutic strategy exploration of *F. nucleatum*-related diseases.

## Introduction

*Fusobacterium nucleatum* (*F. nucleatum*), which is an anaerobic, Gram-negative commensal bacterium from the Fusobacteriaceae family, has attracted much attention as a critical member of both the oral and gut microbiota in recent years (Signat et al., 2011; Kostic et al., 2013; Han, 2015; Yu et al., 2017a; Brennan and Garrett, 2019; Wong and Yu, 2019). Increased abundance of *F. nucleatum* is reported to be associated with several human diseases, including oral infections, adverse pregnancy outcomes and gastrointestinal diseases such as inflammatory bowel disease (IBD) and colorectal cancer (CRC) (Strauss et al., 2011; Han, 2015; Brennan and Garrett, 2019; Liu et al., 2020).

*F. nucleatum* is suggested to form aggregates with other oral pathogens and lead to periodontal diseases (Bolstad et al., 1996; Feuille et al., 1996). Furthermore, the chance of isolating *F. nucleatum* from patients with gastrointestinal diseases is significantly higher than from healthy controls (Strauss et al., 2011). *F. nucleatum* is distinctly enriched in the feces of inflammatory bowel disease (IBD) patients, and a higher copy number of *F. nucleatum*-specific genes was detected in CRC tissues than in para-tumor normal tissues (Kostic et al., 2012; Liu et al., 2020). Transcriptomics analysis confirmed the enrichment of *F. nucleatum* in CRC tissues (Castellarin et al., 2012). *F. nucleatum* participates in the occurrence and development of CRC in a variety of ways, such as promoting the proliferation and metastasis of cancer cells, regulating the tumor microenvironment of CRC, interfering with the metabolic microenvironment and inhibiting immune cell activity (Kostic et al., 2013; Szabo et al., 2013; Rubinstein et al., 2013b; Gur et al., 2015; Mima et al., 2015; Abed et al., 2016; Yang et al., 2017; Chen et al., 2018; Xue et al., 2018; Chen et al., 2020a, 2020b). In addition, colonization of *F. nucleatum* in breast cancer tissue was also found to promote tumor growth and metastasis (Parhi et al., 2020).

Considering the pathogenicity of *F. nucleatum*, regulating the abundance of *F. nucleatum* in the microbiota could be a novel approach to intervening in the development of related diseases. Strategies that aim to decrease the number of *F. nucleatum* include antibiotic intervention, probiotic intervention, inhibitors of *F. nucleatum* surface proteins and phage therapy (Zhu et al., 2010; Rubinstein et al., 2013a; Yu et al., 2015; Jang et al., 2016; Bullman et al., 2017; Wu et al., 2018; Kabwe et al., 2019; Zheng et al., 2019; Dong et al., 2020; Chen et al., 2020c). Among these, bacteriophage therapy, which is believed to precisely target host bacteria, has great potential for applications. Previous studies have mainly focused on the bactericidal activity of phages against multidrug-resistant bacterial infections, which act as antibiotic alternatives. Since specific bacteria were found to play roles in promoting the development of noninfectious diseases, phages have emerged as an excellent therapeutic option for these diseases based on their stringent host specificity. A study reported a strategy of phage mediated gut microbiota modulation to enhance response of colon tumors to the first-line chemotherapy drug irinotecan. A phage with specific antibacterial activity was screen out from human saliva, the phage showed great selectivity to tumor tissues *via* targeting *F. nucleatum* in CRC. This property was designed to help deliver chemotherapy drugs to tumors (Zheng et al., 2019). In another study, a specifically customized *F. nucleatum*-binding M13 phage with AgNP assembled on its surface capsid protein (M13@Ag) achieved specific clearance of *F. nucleatum* and reconstruction the tumor-immune microenvironment (Dong et al., 2020). In addition, phage-targeted therapy has also been tried in the treatment of other non-infectious diseases. Four phages selected from sewage were used to selectively kill the cytolytic *E. faecalis* for treating ethanol-induced liver injury and steatosis in germ-free mice that have transplanted with the fecal microbiota of cytolysin-positive patients (Duan et al., 2019).

Bacteriophage therapy has exclusive advantages. Since there is a “predator–prey” dynamic relationship between phages and their host bacteria which means phages must be symbiotic with host bacteria, so phages might be isolated from a wide range of environments wherever host bacteria are exist. Furthermore, phage therapy is relatively safe for clinical use. Bacteriophages can penetrate the blood–brain barrier and will be cleared from the bloodstream and organs in a short period of time if host bacteria are absent (Geier et al., 1973; Barrow and Soothill, 1997). In addition, phages only specifically attack host bacteria without affecting mammalian cells (Chan et al., 2013). Some phase I and phase II clinical trials of phage therapy for the treatment of multidrug-resistant bacteria have shown its safety in humans (Rhoads et al., 2009; Wright et al., 2009; Jault et al., 2019). It is worth noting that the application of bacteriophages may also enhance immune activity, which has a potential synergistic effect with tumor immunotherapy, as phage components can be regarded as pathogen-associated molecular patterns (PAMPs) which may activate the immune system. Thus, exploring the application possibilities of *F. nucleatum* bacteriophages is the objective of our study.

The existing research on *F. nucleatum* bacteriophages are particularly limited and only seven isolates have been reported (Machuca et al., 2010; Cochrane et al., 2016; Kabwe et al., 2019; Zheng et al., 2019). In this study, five *F. nucleatum* phages were screened out and identified from various clinical samples. Their genomic, morphological and biological characteristics were further studied to provide a basis for subsequent exploration of their therapeutic effects on *F. nucleatum*-related diseases.

## Materials and methods

### Bacterial strains and culture conditions

A total of 11 strains of *F. nucleatum* bacteria were purified and used in this study, of which eight were isolated from saliva samples of healthy adults and one from patients with periodontitis. The samples for isolation of *F. nucleatum* should be sealed into AnaeroPack-Anaero bags as soon as possible after sampling. All the samples were collected from Shanghai Ninth People’s Hospital affiliated with Shanghai Jiao Tong University and Renji Hospital affiliated with Shanghai Jiao Tong University. The other two strains are standard strains *F. nucleatum* ATCC 25586 and ATCC 23726, both of which were provided by Renji Hospital affiliated with Shanghai Jiao Tong University. More detailed information is listed in Table 1. All screened *F. nucleatum* strains were identified by MALDI-TOF MS and 16S rRNA sequencing. The 16S rRNA genes in bacterial genomes were amplified with the forward primer 27F 5’-AGAGTTTGATCCTGGCTCAG-3′ and reverse primer 1492R 5’-GGTTACCTTGTTACGACTT-3′. The PCR program used for the 16S rRNA gene was as follows: initial denaturation for 5 min at 94°C; 30 cycles of 94°C for 1 min, 55°C for 1 min, and 72°C for 1 min 30 s; and a final extension at 72°C for 8 min.

**Table 1:** Lytic spectra of JD-Fnp1 ~ JD-Fnp5 to 11 *F. nucleatum* strains.

Bacterial strains	Sources	Phages*				
		JD-Fnp1	JD-Fnp2	JD-Fnp3	JD-Fnp4	JD-Fnp5
JD-Fn1	Saliva (periodontitis patient)	+	+	+	+	+
JD-Fn2	Saliva (healthy people)	+	−	−	+	−
JD-Fn3	Saliva (healthy people)	−	−	−	−	−
JD-Fn4	Saliva (healthy people)	−	−	−	−	−
JD-Fn5	Saliva (healthy people)	−	−	−	−	−
JD-Fn6	Saliva (healthy people)	−	−	−	−	−
JD-Fn7	Saliva (healthy people)	−	−	−	+	−
JD-Fn8	Saliva (healthy people)	−	+	+	+	−
JD-Fn9	Saliva (healthy people)	−	−	−	−	−
*F. nucleatum* ATCC 25586	Lab	−	−	−	+	−
*F. nucleatum* ATCC 23726	Lab	−	−	−	+	+

* Absence of phage plaques (−).

Presence of phage plaques (+).

All *F. nucleatum* strains were routinely cultured in predeoxygenated brain-heart infusion (BHI) liquid medium at 37°C under anaerobic environment (10% H_2_, 10% CO_2_, 80% N_2_). After resuscitation from frozen preservation, single colonies of each strain were picked and cultured in BHI medium for 12 h, inoculated into 6 ml fresh BHI culture medium at a 2% inoculation ratio, and then cultured anaerobically at 37°C before use. The growth of the bacteria was monitored by measuring the optical density (OD) at 600 nm. BHI plates (1.1% agar, w/v) or BHI soft agar (0.4% agar, w/v) were also used when needed. The liquid cultures from the late logarithmic stage were collected and stored at −80°C after mixing with an equal volume of autoclaved 40% glycerol for preservation.

### Isolation of *Fusobacterium nucleatum* bacteriophages

Three hundred saliva samples from healthy people or patients with periodontitis and 10 fecal samples from patients with CRC were collected for bacteriophage isolation. All the samples were collected from Shanghai Ninth People’s Hospital affiliated with Shanghai Jiao Tong University and Renji Hospital affiliated with Shanghai Jiao Tong University. The saliva samples and fecal samples should be quickly sealed into AnaeroPack-Anaero bags after sampling. A CaCl_2_ solution to a final 5 mM was added to the prefiltered samples, and the mixture was centrifuged at 4000 × g for 15 min at room temperature. Then, the supernatant was filtered through a 0.22 μm pore size filter, and the filtrate was stored at 4°C for short-term preservation. To enrich the bacteriophages in the sample, 3 × BHI liquid medium was added to the tube containing the filtrate, and then *F. nucleatum* in logarithmic growth phase was added. The mixture was placed in an anaerobic incubator for 48 h after mixing thoroughly. After that, the enriched solution was centrifuged at 4000 × g for 10 min, and the supernatant was filtered through a 0.22 μm pore size filter again. The filtered solution was diluted in gradient, and the diluent was co-incubated with the mixed host strains growing in the logarithmic phase for 5 min. Then, the double-layer agar plate method was used to screen the phage (Cui et al., 2017). The plates were cultured in an anaerobic incubator at 37°C and the plaque was checked every 12 h. After the phage was initially found as a plaque in the lawn, the plaques in the upper layer agar were picked with a sterile syringe needle and then added to Sodium Chloride-Magnesium sulphate buffer [SM buffer, (Tris-Mg^2+^ Buffer) 50 mM Tris–HCl (pH 7.5), 100 mM NaCl, 8 mM MgSO_4_, 0.01% Gelatin (v/w))] before placing on a shaker to elute. The eluent was applied to a double-layer agar plate to propagate the phages. After observing the appearance of plaques on the plate again, a large and transparent single plaque was collected for further elution. After repeating the above process three times, the purified phage solution was obtained and stored at 4°C.

### Phage purification, amplification, and morphological characterization

The bacteriophages and candidate bacteria were mixed and cultured in double-layer plates under an anaerobic environment at 37°C for 48 h until the plaques could be observed. SM buffer was added to the plates, and then the plates were placed at 4°C overnight. The eluate was absorbed by an aseptic syringe and filtered through 0.22 μm pore size filter. The phage particles in the filtrate were precipitated using polyethylene glycol 8000 (PEG8000) with final concentration 10% (w/v) by centrifugation at 8,500 rpm for 25 min. The pellets were re-suspended in 1 ~ 2 ml SM buffer, and PEG8000 was removed by adding the equal volume of chloroform, and the mixture was centrifuged at 4,000 g/min for 15 min. Then added CsCl (0.5 g/ml) to the collected supernatant which contained high concentrations of phage. The phages were then purified by ultracentrifugation at 4°C, 140,000 × g for 4 h in a CsCl gradient (1.33, 1.45, 1.50, 1.70 g/cm^3^) in SM buffer. The band containing the enriched phages was taken out using a syringe, and finally dialyzed with SM buffer. The purified bacteriophage fluid was stored at 4°C.

An appropriate amount (~10^9^ PFU/mL) of phage solution was taken and dropped on a carbon-coated copper mesh to prepare the electron microscope sample. After the phage samples were negatively stained with 2% (w/v) phosphotungstic acid for 2 ~ 3 min, the samples could be used for observation. Talos L120C transmission electron microscope (TEM) was used to observe the morphological features of JD-Fnp1 ~ JD-Fnp3. Tecnai G2 Spirit Biotwin biological transmission electron microscope was used to observe the morphological features of JD-Fnp4 ~ JD-Fnp5.

### Determination of the host bacteria

The host spectrum of the bacteriophage was determined by the spot test method. Each strain of the candidate *F. nucleatum* was mixed with 0.7% soft agar before application to BHI plates as the upper layer, and the double-layer plates were divided into five equal parts in advance. Ten microliters of a dilution series of suspension (10^2^ ~ 10^6^ PFU/mL) of the five bacteriophages was spotted on one part of the plates above. Three replicate plates were prepared for each strain. The plates were cultured in an anaerobic incubator at 37°C for 48 h until the plaques were observed. The host bacteria were confirmed based on the appearance of the bacteriophage plaques on the plates.

### Determination of the adsorption rate

The adsorption curve detection of the isolated phages was carried out according to the method of Cui et al. with some modifications (Cui et al., 2017). In short, the host bacteria were cultured to the logarithmic growth phase, and purified bacteriophage was adjusted to the multiplicity of infection (MOI) value equal to 0.01. The mixture of bacteria and bacteriophage was placed in a 37°C anaerobic incubator, and the mixture was taken at different time points. The mixture was centrifuged at 13,400 × g at 4°C for 1 min. After centrifugation, the supernatant was mixed with the host bacterial solution for double-layer plate enrichment. The titer of the bacteriophage in the supernatant was determined by counting the plaques on the plates. The bacteriophage adsorption curve was drawn according to the bacteriophage titers measured at different time points.

### Determination of one-step growth curve of phage JD-Fnp1 ~ JD-Fnp5

The modified one-step growth curves of the five phage isolates were determined by monitoring increase in the number of phage particles during a replicative cycle. Briefly, five milliliters of host bacteria *F. nucleatum* JD-Fn1 cultured to logarithmic growth phase was centrifuged at 10,000 × g for 8 min, and the precipitate was resuspended in BHI liquid medium. The bacteriophage was added to the bacterial resuspension with MOI = 0.01. The mixture of bacteriophage and bacteria was placed in an anaerobic incubator at 37°C for 20 min. After incubation, the mixture was centrifuged at 10,000 × g for 5 min, and the supernatant containing non-adsorbed bacteriophage was discarded. The precipitate was placed in an anaerobic incubator after resuspension in BHI medium. Results are reported as the increase of phage titer calculated by enumerating the average PFU/mL burst into the medium. The one-step growth curve of the bacteriophage was drawn according to the titers obtained.

### Stability evaluation of phages JD-Fnp1 ~ JD-Fnp5

Stability of five phage isolates were conducted to evaluate their abilities to withstand harsh conditions. To study the thermal stability, the bacteriophages were diluted with SM buffer and incubated in a water bath at different temperatures (4°C, 37°C, 40°C, 50°C, 60°C and 70°C) for 1 h. After that, the double-layer plates with the host bacteria and five phage isolates with different temperature treatment were mixed and laid immediately, then the titers were examined and compared with control groups. The control temperature of JD-Fnp1 ~ JD-Fnp4 is 37°C, and the control temperature of JD-Fnp5 is 4°C which the JD-Fnp5 has the best stability. For pH stability, each of the phage isolate were added to SM buffers with pH values ranging from 4 to 10, then the phage titers were determined same as above mentioned after incubation in a water bath at 37°C for 1 h.

### Growth curve of host bacteria infected by phages JD-Fnp1 ~ JD-Fnp5

To evaluate the *in vitro* bactericidal activity of five phage isolates, the growth curve of host bacteria *F. nucleatum* JD-Fn1 was determined at different MOIs. Six milliliter of JD-Fn1 cultures from the early logarithmic stage was incubated with phages at different MOIs (JD-Fnp1 and JD-Fnp3 used MOIs = 0, 0.1, 0.01, 0.001; JD-Fnp2 and JD-Fnp5 used MOIs = 0, 1, 0.1, 0.01; JD-Fnp4 used MOIs = 0, 1,000, 100, 10) at 37°C under anaerobic environment. OD_600_ values of *F. nucleatum* JD-Fn1 was measured with spectrophotometer every 2 h during 22 h or 32 h. The growth curve of the bacteria was completed according to the OD_600_ values at different time points.

### Genome sequence analysis and safety assessment

The five phages screened in this study were all transferred to the National Human Genome Southern Research Center (Shanghai, China) for whole-genome sequencing. DNA from the phages was extracted using the QIAamp DNA Mini Kit (Qiagen, Venlo Netherlands), following manufacturer’s instructions. Purified phage DNA was used for library prep using TruSeqTM DNA Sample Prep Kit - Set A (illumina, United States). Preparation of PE libraries using TruSeq PE Cluster Kit (illumina, United States) then. Sequencing was performed with the Illumina NovaSeq platform with a read length of 151 bp. High quality reads of five phage isolates acquired after whole genome sequencing are 12,235,785 (JD-Fnp1), 8,519,996 (JD-Fnp2), 4,701,760 (JD-Fnp3), 5,616,092 (JD-Fnp4) and 11,781,305 (JD-Fnp5).

Newbler v2.8 was used to assemble the data with its default settings. ORFs were predicted using RAST^1^ (Aziz et al., 2008) with default settings, and annotated using BLASTp^2^ to explore potential function.

After obtaining the complete genome sequences of JD-Fnp1 ~ JD-Fnp5, we performed genome-based classification of JD-Fnp1 ~ JD-Fnp5 using GRAViTy^3^ (Aiewsakun et al., 2018; Turner et al., 2021b) and vConTACT v.2.0 (Bin Jang et al., 2019; Turner et al., 2021b). When receiving result files generated by GRAViTy (Aiewsakun et al., 2018; Turner et al., 2021b), we visualized and annotated the results using iTOL^4^ (Letunic and Bork, 2021).

The safety of the phage was evaluated based on genome sequence analysis. Databases ARDB^5^ (Liu and Pop, 2009) and VFDB^6^ (Chen et al., 2005) were used to analyze antibiotic resistance genes and virulence factors in the genomes of the five bacteriophage isolates, respectively. The whole genome sequences were compared against the database to explore whether the phage carried related genes. Genes with more than 70% coverage and a minimum identity of 40% were screened.

The phylogenetic tree of JD-Fnp1 ~ JD-Fnp5 with their close relatives was constructed with VipTree^7^ (Nishimura et al., 2017) based on whole genome sequences. The results of VipTree (Nishimura et al., 2017) were used as the reference of genome-based classification of phage JD-Fnp1 ~ JD-Fnp5 as well.

Similarity analysis of the JD-Fnp1 ~ JD-Fnp5 genomes was performed with blastn (BLAST v2.2.31+). We referred to two excellent articles in the post-sequencing analysis and annotation process (Shen and Millard, 2021; Turner et al., 2021a).

In order to further explore the similarity between the fusobacterium-related phage sequences in metagenomic database and the genome of our isolates, we used all gene encoding proteins of JD-Fnp1 ~ JD-Fnp5, FNU1, φFunu1 and metagenomics assemblies from IMG/VR v3^8^ (Roux et al., 2021) for comparison with Roary (Page et al., 2015) with different *i* value (*i* = 60; *i* = 70; *i* = 80). Other parameters used in protein Roary-based analysis are -cd 99 and otherwise default parameters.

We ran conserved domain search for JD-Fnp1 ~ JD-Fnp5, FNU1, φFunu1, and metagenomics assemblies from IMG/VR v3 with CDD^9^ (Lu et al., 2020), two datasets were obtained (one for *e* = 0.01 and one for *e* = 0.0001). Then we created presence/absence heatmaps with the default parameter value based on the results for two datasets (one for *e* = 0.01 and one for *e* = 0.0001), the heatmap is based on the presence or absence of the CDD in the phage-related genome, cluster genomes and cluster domains, sort genomes and domains accordingly.

## Results

### Morphological characteristics of *Fusobacterium nucleatum* bacteriophages JD-Fnp1 ~ JD-Fnp5

**Figure 1:**
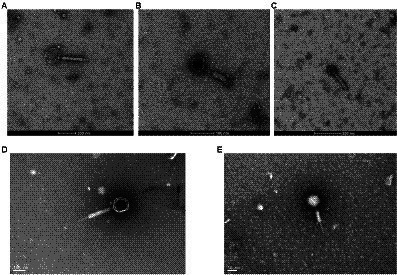
TEM micrograph of phages JD-Fnp1 ~ JD-Fnp5. **(A)** JD-Fnp1. The length and width of the head are 155 ± 5 nm and 140 ± 5 nm respectively, and the length of its retractable tail is 335 ± 5 nm. Magnification: × 36 k. **(B)** JD-Fnp2. The length of its head is 60 ± 2 nm, and the width is 50 ± 3 nm. The retractable tail is 130 ± 5 nm in length. Magnification: × 73 k. **(C)** JD-Fnp3. It has a head of the polyhedron with a radial length of 55 ± 3 nm, and a retractable tail with a length of 135 ± 2 nm. Magnification: × 54 k. **(D)** JD-Fnp4. The diameter of the capsid head and the length of the flexible tail are about 130 ± 5 nm and 300 ± 4 nm. Magnification: × 98 k. **(E)** JD-Fnp5. The diameter of the capsid head and the length of the flexible tail are 61 ± 2 nm and 144 ± 5 nm. Magnification: × 150 k. These measurements were taken on 10 different particles.

Five isolates of *F. nucleatum* bacteriophages were screened from human samples, four of which (JD-Fnp1 ~ JD-Fnp4) were isolated from saliva samples of healthy people, and one (JD-Fnp5) was derived from a fecal sample of a patient with CRC. We can conclude the morphotypes of the phage isolates we acquired. The microscopic images of JD-Fnp1 ~ JD-Fnp5 were characterized by transmission electron microscopy (TEM) and are displayed in Figure 1. The morphological identification showed that JD-Fnp1 ~ JD-Fnp5 are all myoviruses in morphotype. The head of JD-Fnp1 has a prolate geometry with a length of 155 ± 5 nm and a width of 140 ± 5 nm, and the length of its tail is 335 ± 5 nm. The head of JD-Fnp2 has a prolate geometry, elongated only in one direction, which is similar to JD-Fnp1. Electron micrographs show that the length of its head is 60 ± 2 nm, and the width is 50 ± 3 nm. The tail of JD-Fnp2 is 130 ± 5 nm in length. JD-Fnp3 has a head of the polyhedron with a radial length of 55 ± 3 nm, and a retractable tail with a length of 135 ± 2 nm. JD-Fnp4 has a polyhedral head with a diameter of 130 ± 5 nm and a retractable tail with a length of 300 ± 4 nm. For JD-Fnp5, its polyhedron head diameter is 61 ± 2 nm, and its contractile tail is 144 ± 5 nm long.

### The lytic spectra of JD-Fnp1 ~ JD-Fnp5

The results showed that all five bacteriophages were able to lyse the JD-Fn1 strain, but their lysis ability of other *F. nucleatum* strains was not identical (Table 1). Among them, JD-Fnp4 has the widest host spectrum and lysed 6/11 of the tested strains, including *F. nucleatum* ATCC 25586 and *F. nucleatum* ATCC 23726. The other four phages could only produce plaques on 2/11 of the tested strain lawns, including JD-Fn1.

### Infection profile of phage JD-Fnp1 ~ JD-Fnp5

**Figure 2:**
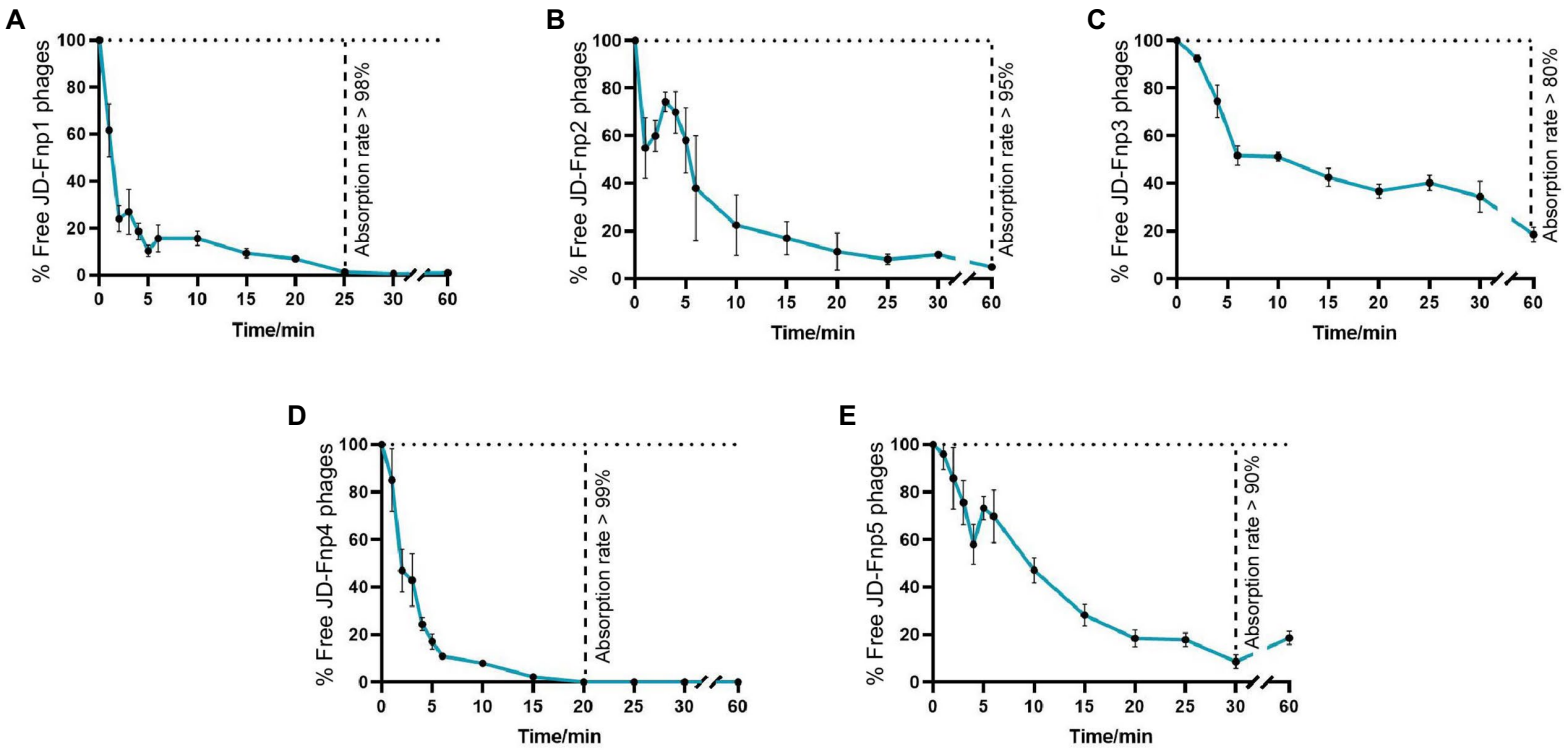
The adsorption profile of phages JD-Fnp1 ~ JD-Fnp5. **(A)** JD-Fnp1; **(B)** JD-Fnp2; **(C)** JD-Fnp3; **(D)** JD-Fnp4; **(E)** JD-Fnp5. The x-axis shows the incubation time of phages with host bacteria, and the y-axis shows the percentage of free phage particles. Data are from three times independent experiments.

The results (Figure 2) showed that JD-Fnp4 reached the highest adsorption rate in the shortest time, followed by JD-Fnp1. After co-incubation with host strain JD-Fn1 for 5 min, more than 80% of the phage particles adhered to the bacteria, while after 20 min, more than 98% of phages remained attached to the host, and the ultimate adsorption rate was as high as 98%. It is also worth noting that half of the phages attached to the host cells within approximately 2 min, demonstrating a high affinity between phages and the host. In addition, JD-Fnp5 reached a final adsorption rate of 90% after co-incubation with JD-Fn1 for 30 min. JD-Fnp2 and JD-Fnp3 achieved the highest adsorption rates of 95 and 80% after 60 min co-incubation with JD-Fn1.

**Figure 3:**
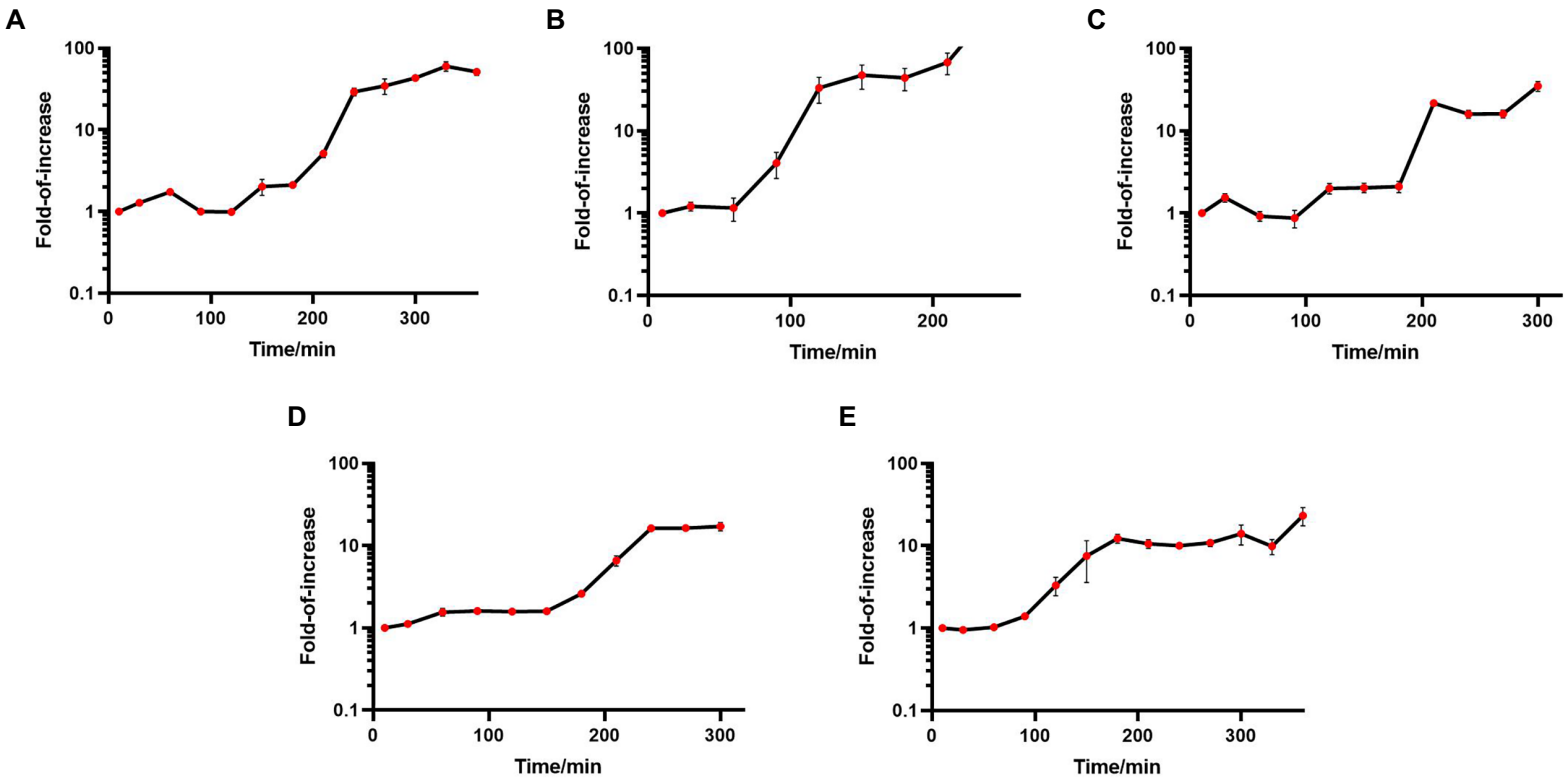
The one-step growth profile of phages JD-Fnp1 ~ JD-Fnp5. **(A)** JD-Fnp1; **(B)** JD-Fnp2; **(C)** JD-Fnp3; **(D)** JD-Fnp4; **(E)** JD-Fnp5. The x-axis shows the incubation time of phages with host bacteria; the y-axis shows the fold of increase of phage titers at different time points. Data are from three times independent experiments.

The one-step growth curve is an indicator of bacteriophage growth. The results are shown in Figure 3. Among the five phages, the latency periods of JD-Fnp1 and JD-Fnp3 were both approximately 180 min, but the rise period of JD-Fnp1 was longer, approximately 150 min, which was approximately five times that of JD-Fnp3. During the whole growth process, JD-Fnp1 could release approximately 60 progeny particles, which is the largest number of progeny among the five phages. The number of progenies produced by JD-Fnp3 was approximately 20 times the number of parent phages. The latency period of JD-Fnp4 was 150 min, followed by a 150 min ~ 240 min rise period. The number of JD-Fnp4 phage particles peaked at 240 min after infection, and then the growth of JD-Fnp4 entered the plateau period. The number of progeny produced during the whole growth process was approximately 17 times the number of parent phages. The latency periods of JD-Fnp2 and JD-Fnp5 were shorter, both approximately 60 min, and their rise periods are 90 min and 120 min, which can produce approximately 45 and 13 progeny particles, respectively.

### Stability of phage JD-Fnp1 ~ JD-Fnp5

**Figure 4:**
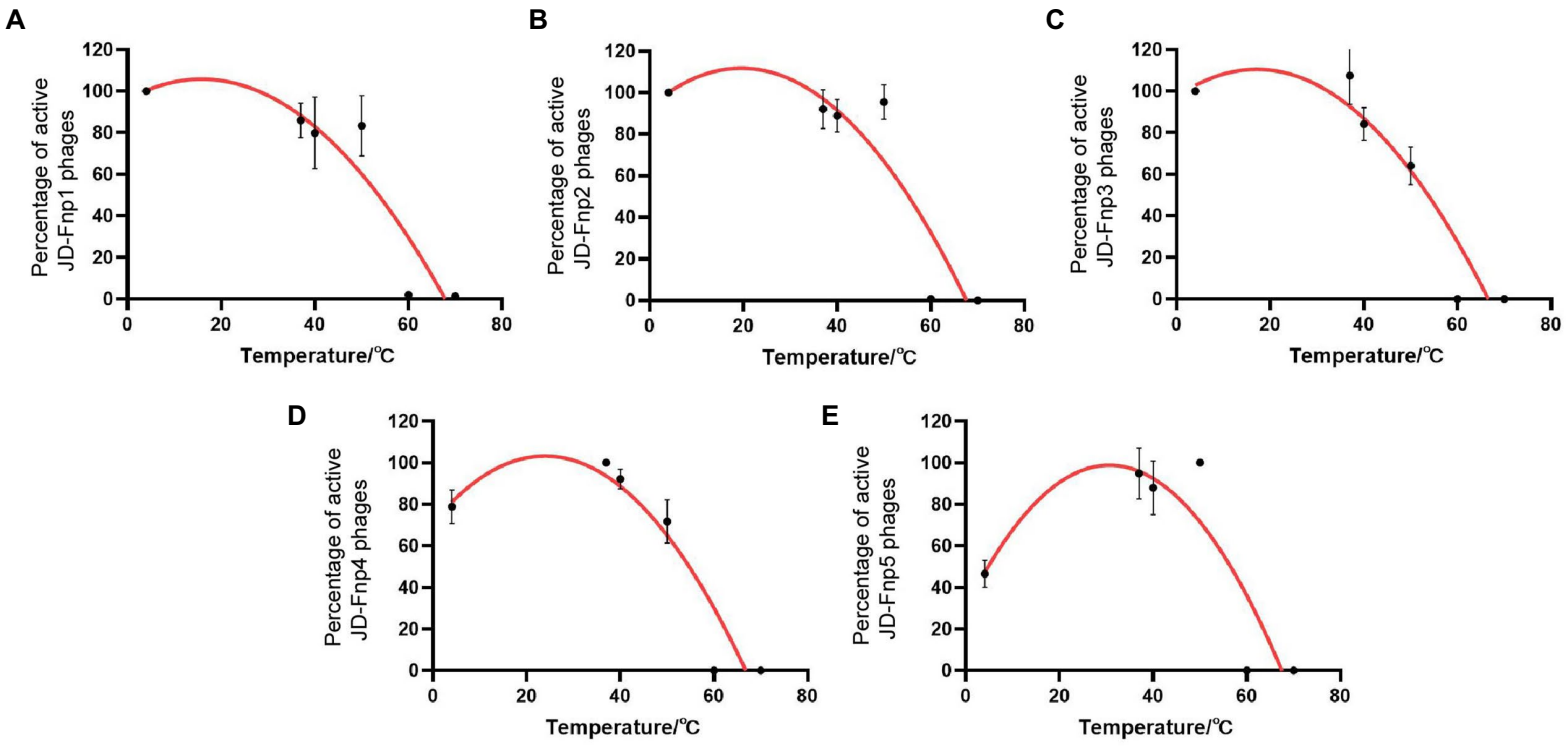
Thermal stability of phages JD-Fnp1 ~ JD-Fnp5. **(A)** JD-Fnp1; **(B)** JD-Fnp2; **(C)** JD-Fnp3; **(D)** JD-Fnp4; **(E)** JD-Fnp5. The x-axis represents temperature range; the y-axis represents the relative titer of JD-Fnp phage to the titer at 37°C or 4°C (The control temperature of JD-Fnp1 ~ JD-Fnp4 is 37°C, and the control temperature of JD-Fnp5 is 4°C that the phage has the best stability); Percentage of active JD-Fnp phages = (N/N0) × 100, where N is the number of viable phages after 1 h of incubation and N0 was the initial number of phages. All experiments were repeated in triplicate.

**Figure 5:**
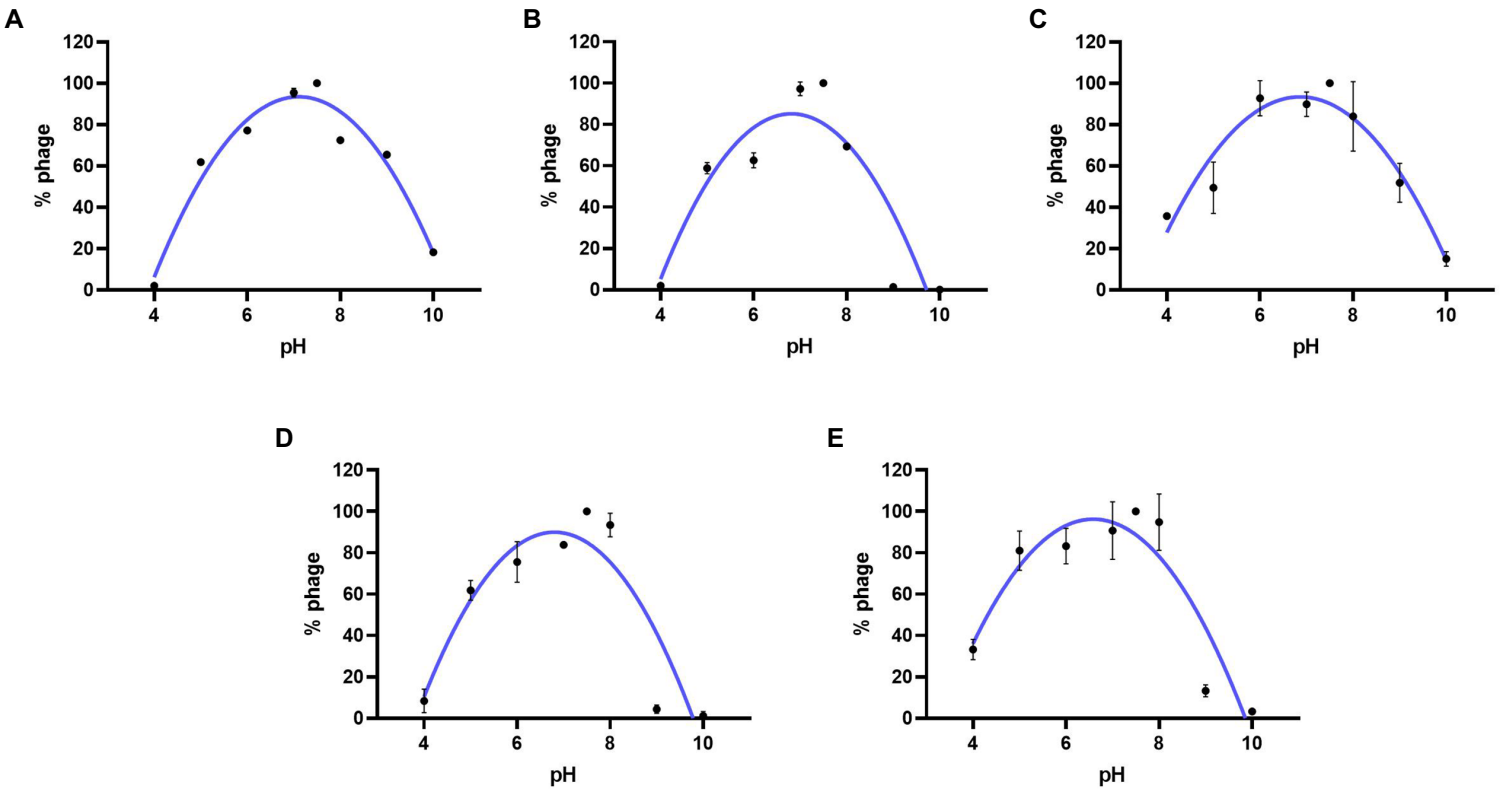
pH stability of phages JD-Fnp1 ~ JD-Fnp5. **(A)** JD-Fnp1; **(B)** JD-Fnp2; **(C)** JD-Fnp3; **(D)** JD-Fnp4; **(E)** JD-Fnp5. The x-axis represents the pH range; the y-axis shows the percentage of the active phage titers in different pH values to that in pH 7.5. All experiments were repeated in triplicate.

The results of the thermal stability and pH stability tests are shown in Figures 4, 5. The activity of JD-Fnp1 remained high level after incubation at temperatures lower than 50°C but was almost completely lost at temperatures above 60°C. JD-Fnp1 was tolerant to pH ranges from 5 to 9. The temperature stability of phage JD-Fnp2 were relatively similar to that of JD-Fnp1, and the pH stability range of JD-Fnp2 was pH 5 to 8. More than 50% of JD-Fnp3 maintained good activity after incubation at 50°C or below, and for pH values, the active range was between 5 and 9. JD-Fnp4 were active infectious particles between 4°C and 50°C, but after incubation at temperatures above 60°C, almost all phages were inactivated or lost their infectivity. More than 50% of the JD-Fnp4 phage remained stable under conditions with a pH value of 5 to 8. The thermal stability of JD-Fnp5 is significantly different from those of the other four phages. JD-Fnp5 remained active between 37°C and 40°C, and as the temperature decreased, the stability decreased by approximately 50% at 4°C; the pH stability range of JD-Fnp5 was pH 5 to 8.

### *In vitro* bactericidal activity of JD-Fnp1 ~ JD-Fnp5 against host *Fusobacterium nucleatum* strains

**Figure 6:**
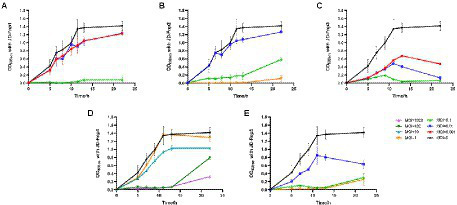
*In vitro* bactericidal activity of JD-Fnp1 ~ JD-Fnp5 with host bacteria JD-Fn1. **(A)** JD-Fnp1; **(B)** JD-Fnp2; **(C)** JD-Fnp3; **(D)** JD-Fnp4; **(E)** JD-Fnp5. *F. nucleatum* strain JD-Fn1 was infected by JD-Fnp phages with different MOI. The x-axis represents the co-culture time of JD-Fnp phage and JD-Fn1; the y-axis represents the change of OD_600_ in the mixture. Data are displayed as the means ± SD (error bars) from three independent experiments.

All five bacteriophages were applied to infect the host bacterium JD-Fn1 with a series of multiplicity of infection (MOI) values at 37°C under anaerobic conditions. JD-Fnp4, the phage with the broadest host spectrum, required MOI = 100 or MOI = 1,000 to achieve a significant inhibitory effect on JD-Fn1, but the growth of JD-Fn1 could be almost completely inhibited during 13 h of incubation (Figure 6). The *in vitro* bactericidal activities of JD-Fnp1, JD-Fnp2, JD-Fnp3 and JD-Fnp5 were similar; when the MOI was equal to or greater than 0.1, the phages significantly inhibited the growth of the host bacterium JD-Fn1 (Figure 6).

After co-incubation of JD-Fnp4 with ATCC 25586 for 13 h, the MOI = 1,000 group achieved a significant inhibitory effect compared with the control group, and the OD_600_ value of the bacteria decreased rapidly (Supplementary Figure S1). The results were similar for ATCC 23726; after JD-Fnp4 was co-incubated with the bacteria for 18 h, the inhibitory effect of the MOI = 1,000 group was remarkable (Supplementary Figure S1).

### Genomic analysis of JD-Fnp1 ~ JD-Fnp5

**Figure 7:**
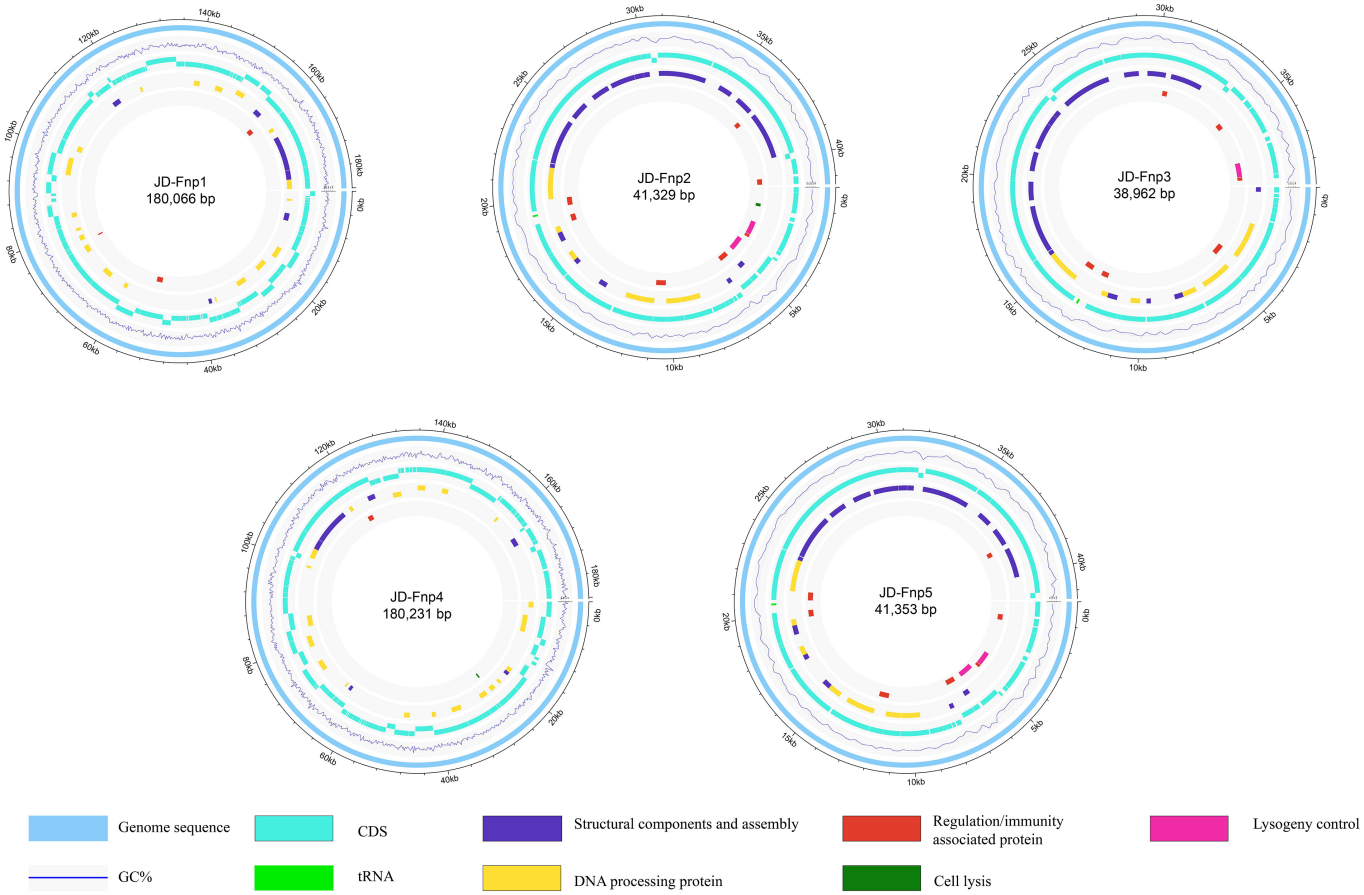
Complete genome sequences of JD-Fnp1 ~ JD-Fnp5. The functional modules were assigned based on gene annotation and genomic organization and are shown in different colors.

The sequencing results showed that the genomes of JD-Fnp1 ~ JD-Fnp5 are all double-stranded DNA, and the basic information is shown in Figure 7 and Table 2. The genome sizes of JD-Fnp1 and JD-Fnp4 are 180,066 bp and 180,231 bp respectively, while the other three phages are smaller, 41,329 bp (JD-Fnp2), 38,962 bp (JD-Fnp3) and 41,353 bp (JD-Fnp5), respectively. The GC% contents of the JD-Fnp1 ~ JD-Fnp5 are similar (30% ~ 32%), and the percentage of coding sequences (CDSs) of the JD-Fnp1 ~ JD-Fnp5 genomes are of 92% ~ 95% similarly (Table 2).

**Table 2:** Genomic information of JD-Fnp1 ~ JD-Fnp5.

	JD-Fnp1	JD-Fnp2	JD-Fnp3	JD-Fnp4	JD-Fnp5
Genome type	dsDNA	dsDNA	dsDNA	dsDNA	dsDNA
Length(bp)	180,066	41,329	38,962	180,231	41,353
GC content %	31.51	30.07	30.27	31.11	30.07
CDS	178	72	68	177	72
CDS percent %	93.66	94.13	94.45	92.94	94.13
Virulence factors	0	0	0	0	0
Antibiotic resistance genes	0	0	0	0	0

ORF: Open reading frame.

CDS: Coding sequence.

**Figure 8:**
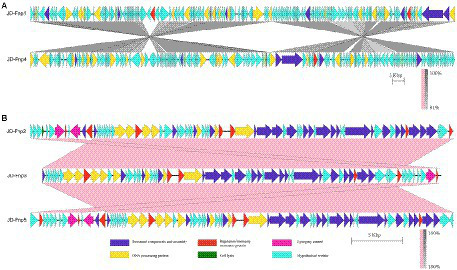
Comparative genomic analysis of JD-Fnp1 ~ JD-Fnp5. **(A)** JD-Fnp1 and JD-Fnp4; **(B)** JD-Fnp2, JD-Fnp3 and JD-Fnp5. Analysis was performed with blastn (BLAST v2.2.31+) at DNA level. The nucleotide identity of the homologous regions (percentage) is shown as a color gradient, the pink bar represents for forward blast hits and the gray bar represents for inverted blast hits; the scale is shown below. Known functional homologous genes are shown below.

JD-Fnp1 and JD-Fnp4, which have relatively larger genomes, the percentage of ORFs which could be annotated were less than the other three. JD-Fnp1 contains 178 putative ORFs of which 16.85% (30/178) had known function. JD-Fnp4 contains 177 putative ORFs of which 16.38% (29/177) had known function. For JD-Fnp2, its genome is composed of 72 predicted ORFs, 54.17% (39/72) of which had known function. The genome of JD-Fnp3 comprised 68 ORFs of which 51.47% (35/68) had known function. JD-Fnp5 genome is composed of 72 predicted ORFs, 52.78% (38/72) of which had known function. For these 5 bacteriophages, we categorized ORFs had known function into five modules: DNA processing, regulation/immunity system, structural components and assembly, cell lysis, and lysogeny control (Figures 7, 8; Table 3; Supplementary Table S1).

**Table 3:** ORFs annotation information of JD-Fnp1 ~ JD-Fnp5 genomes.

	JD-Fnp1	JD-Fnp2	JD-Fnp3	JD-Fnp4	JD-Fnp5
ORFs	178	72	68	177	72
DNA processing*	20	7	8	21	8
Regulation/immunity system*	3	7	6	1	7
Structural components and assembly*	7	22	20	6	21
Cell lysis*	0	1	0	1	0
Lysogeny control*	0	2	1	0	2
Hypothetical protein*	148	33	33	148	34

* The number of ORFs contained in this module.

The results of genome-based taxonomy of phage JD-Fnp1 ~ JD-Fnp5 consistently showed that they all belong to *Myoviridae*, no more taxonomic information was obtained by GRAViTy and vConTACT v.2.0.

In addition, we analyzed the genomic similarity of JD-Fnp1 ~ JD-Fnp5 and found that the identity between JD-Fnp4 and JD-Fnp1 was approximately 90.82%, with a coverage of 93%. The other three phages had genomes size of approximately 40 kb, and any two of them had an identity higher than 99% (Figure 8).

### Safety assessment of JD-Fnp1 ~ JD-Fnp5 and phylogenetic relatedness of the 5 phages with relative bacteriophages

The annoted functional genes of JD-Fnp1 ~ JD-Fnp5 were compared against the antibiotic resistance genes database ARDB and the virulence factors database VFDB. The results showed that no virulence factors or antibiotic resistance genes were predicted in the five phage genomes.

**Figure 9:**
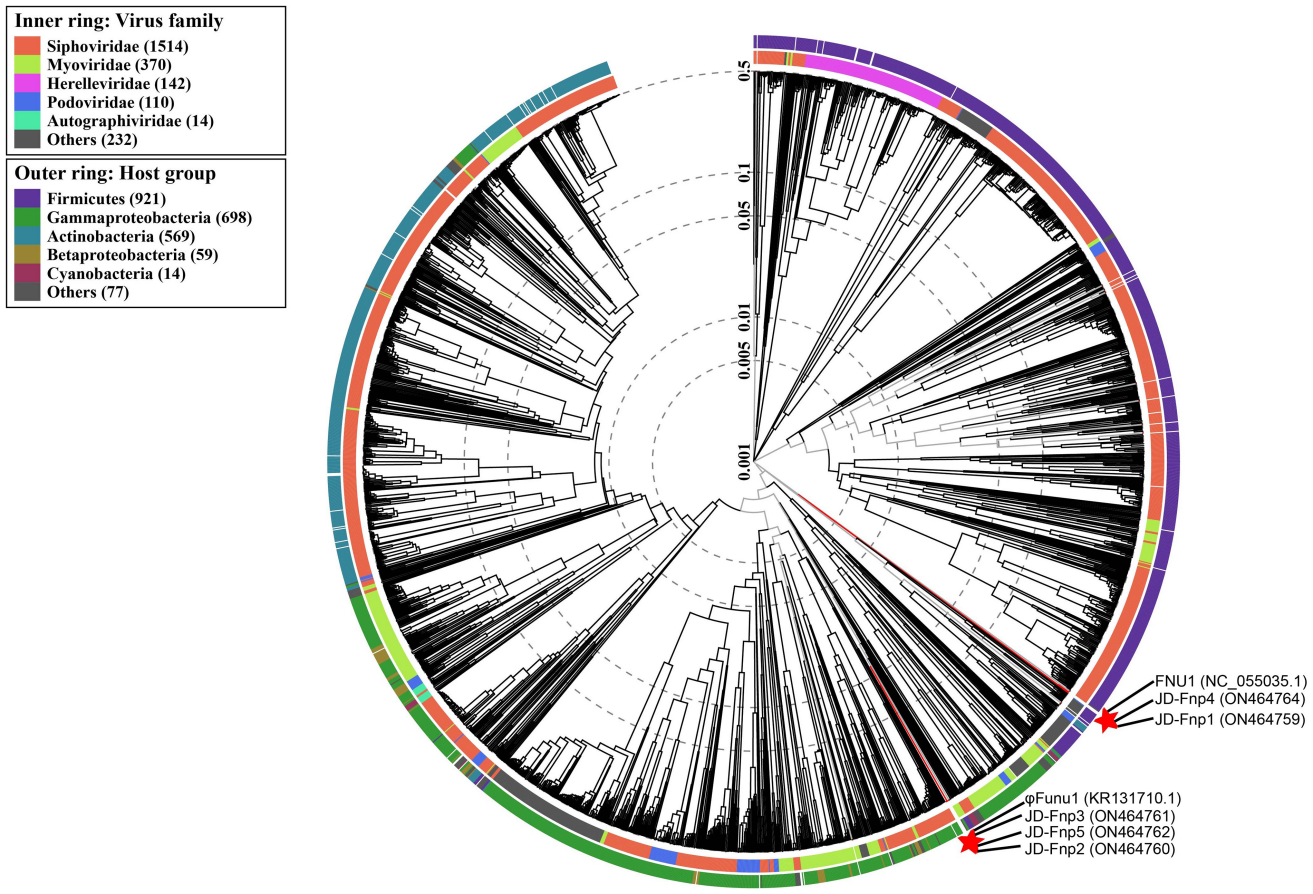
Phylogenetic tree of JD-Fnp1 ~ JD-Fnp5 and relative bacteriophages based on the complete genome sequences. The genome sequences of two *F. nucleatum* phages reported previously were downloaded from NCBI, and all the accession numbers are shown in () following the names of phages. The phylogenetic tree was constructed with VipTree (http://www.genome.jp/viptree) based on whole genome sequences at DNA level.

A phylogenetic tree was constructed based on genome-wide similarities of JD-Fnp1 ~ JD-Fnp5 and that of their close relatives. In the phylogenetic tree (Figure 9), JD-Fnp1, JD-Fnp4 and one *F. nucleatum* bacteriophage FNU1 mentioned in previous articles clustered in the same phylogenetic sub-branch. JD-Fnp2, JD-Fnp3, and JD-Fnp5 were clustered together with another previously reported *F. nucleatum* phage φFunu1.

The results of similarity comparison among JD-Fnp1 ~ JD-Fnp5 with previously reported phages are shown in the Supplementary material. The results of the analysis at the protein level are shown in Supplementary Figure S2 (*i* = 80). It can be concluded from the results that the encoding proteins of JD-Fnp1 and JD-Fnp4 overlap little with other phages, indicating that the similarity is low. Although JD-Fnp2, JD-Fnp3 and JD-Fnp5 partially overlap with some other phages-associated proteins, the similarity among them is not high. And other two results with *i* = 60 and *i* = 70 gave similar results.

The results of the comparison of conserved domains are shown in Supplementary Figure S3 (*e* = 0.0001). From the picture, JD-Fnp1 ~ JD-Fnp5 showed little similarity with other phages in genome level, and less strict cut-off (*e* = 0.01) gave similar results.

## Discussion

Isolating bacteriophages targeting anaerobic bacteria is challenging, information about phage isolates of obligate anaerobes is scarce, and much of what does exist concerns temperate phages. The lack of specialized techniques and the difficulties involved in working with anoxic systems and microorganisms that are difficult to grow hinder the search for these bacteriophages (Hernández and Vives, 2020). However, obligate anaerobic bacteria in the gut accounts for the majority of the biome, so these phages are important due to the impact of their hosts on human and animal health. *F. nucleatum* has attracted much attention due to its association with CRC development; thus, bacteriophage isolates for this bacterial species are considered to have a good application perspective. Only seven isolates of *F. nucleatum* phages have been reported before (Machuca et al., 2010; Cochrane et al., 2016; Kabwe et al., 2019; Zheng et al., 2019). Five isolates of novel lytic bacteriophage were successfully isolated in our study. Moreover, the morphology, biological features and genome sequences of these five isolates were fully characterized. The information on the samples we used and the methods we modified provide a reference for future studies on bacteriophages of anaerobic bacteria.

In regard to the seven isolates of previously reported bacteriophages, the samples used for isolation were two saliva samples (Machuca et al., 2010; Zheng et al., 2019), two dental practice samples, including one drainage sample from dental chairs and one mouthwash sample, and one fecal sample from male C57BL/6 J Apc^Min/+^ mice (Machuca et al., 2010; Kabwe et al., 2019; Zheng et al., 2019). In addition, two lysogenic phages were successfully induced by mitomycin C from *F. nucleatum* subspecies *animalis* strain 7–1 (Cochrane et al., 2016). In the current study, JD-Fnp1 ~ JD-Fnp4 were isolated from saliva samples of healthy people, and JD-Fnp5 was derived from fecal samples of a patient with CRC. Based on these results, we found that the isolation rate of *F. nucleatum* phages from saliva is higher than that from other sources, and saliva samples could be an excellent source for *F. nucleatum* phages mining.

Phages not only live in bacteria but also lyse their host bacteria, which have been described as “predator–prey” dynamic models (Koskella and Brockhurst, 2014). The abundance of bacteriophages is usually in accordance with their host strains. For future clinical application, the ideal sample type for phage and host bacteria isolation is fecal samples from CRC patients. However, since the composition of the gut microbiota is extremely complicated, phage screening is difficult. Saliva samples may act as a perfect alternate due to the existence of a high abundance of *F. nucleatum.* It has been reported that there is a high similarity between the *F. nucleatum* strains isolated from the gut and oral cavity. Our phylogenetic tree showed that JD-Fnp5 was located in the same branch as JD-Fnp2 and JD-Fnp3, which indicated that the source of phage isolation is not related to the genomic relatedness and phylogenetic affinities among different phages. We conclude that saliva samples are a suitable source for phage screening.

It is also worth noting that the samples for isolation should be sealed into AnaeroPack-Anaero bags as soon as possible after sampling to maintain an anaerobic environment to ensure the survival of the host bacteria and prolong the survival time of the phages. When processing the sample, it should also be carried out in an anaerobic environment as much as possible. Then, the processed samples should be quickly mixed with the *F. nucleatum* host bacterial solution and placed in an anaerobic incubator for co-incubation. Due to the slow growth rate of *F. nucleatum*, it usually takes more than 24 h to observe the appearance of plaques after culture, longer than aerobic phages.

Based on the morphological study by transmission electron microscopy (TEM), the morphotype of the isolated phages could be identified. JD-Fnp1 ~ JD-Fnp5 are all myoviruses. For the reported *F. nucleatum* phages, only four isolates were classified, two of which are siphoviruses (Machuca et al., 2010; Kabwe et al., 2019). The other two isolates are myoviruses (Cochrane et al., 2016). These findings indicate a morphological diversity of *F. nucleatum* phages. No significant relationship was observed between the families and the biological features of the phages we isolated.

Nowadays, with the development of sequencing technology and the continuous enrichment of database data, genome-based phage taxonomy appeared to be an important classification strategy in phage-related research (Turner et al., 2021b). Some bioinformatic tools can be used for classifying, clustering, and analyzing phages based on phage genome information. Nevertheless, the researches on the genome of *F. nucleatum* phages are rare in published research. In our study, to determine the genome-based classification of our phage isolates, both GRAViTy and vConTACT v.2.0 were used, and the results from VipTree were used as the reference, the taxonomy was determined by sequence relatedness, most related genomes and genomic similarity of JD-Fnp1 ~ JD-Fnp5 with existed data in database (Nishimura et al., 2017; Aiewsakun et al., 2018; Bin Jang et al., 2019). However, no detailed taxonomic information was got based on these methods, the results only indicated that they all belong to *Myoviridae*. Phages that are evolutionarily similar to our phages or have high identity with our phage genome sequences were not found.

The International Committee on Taxonomy of Viruses (ICTV) is the currently recognized classification system for bacteriophages, which will be updated in time according to current research progress (Turner et al., 2021b). However, “*Siphoviridae*,” “*Myoviridae*,” “*Podoviridae*” have been abolished from the latest ICTV classification system in the latest version (Turner et al., 2021b), but in fact, these family names still exist in the current genome-based classification tools which may due to a delayed update. We attempt to classify JD-Fnp1 ~ JD-Fnp5 based on the ICTV classification system. Unfortunately, we did not find phages with genome similarity to our phages in the NCBI database. We found the only one *F. nucleatum* phage FNU1 in the latest ICTV classification system, and we blasted the genome sequences of our five phage isolates with the FNU1 genome (Kabwe et al., 2019). No significant similarity was found between JD- Fnp1 ~ JD-Fnp5 and FNU1. Here we can only classify JD-Fnp1 ~ JD-Fnp5 as members of *Myoviridae* according to the traditional classification system, as morphologically and genomically they are all classified as so.

The host spectrum determines the application value of phages. For the previously reported isolates, only four of the seven were evaluated for host spectrum, three of which were restricted to *F. nucleatum* but the exact details were limited (Machuca et al., 2010; Kabwe et al., 2019; Zheng et al., 2019). In our study, JD-Fnp4 has the widest host spectrum and can infect 6/11 of the tested isolates, including two *F. nucleatum* standard strains, ATCC 25586 and ATCC 23726, whose pathogenicity has been reported (Castellarin et al., 2012; Yu et al., 2017b; Parhi et al., 2020). ATCC 25586 was significantly enriched in colorectal carcinoma tissues compared to adjacent normal tissues, and it can be successfully applied to CRC animal modeling (Castellarin et al., 2012; Yu et al., 2017b). ATCC 23726 specifically colonizes mouse breast cancer tissues and promotes tumor growth and metastasis (Parhi et al., 2020). The study of biological characteristics confirmed the excellent potential clinical application value of JD-Fnp4. First, JD-Fnp4 remains relatively stable at a normal body temperature of 37°C. The pH stability range of JD-Fnp4 is between pH 6 ~ 8, which indicates that if the application requires oral or intragastric delivery, we can encapsulate phage or use NaHCO_3_ to temporarily neutralize the acidic environment of the stomach (Hsu et al., 2019; Loh et al., 2020).

The biological characteristics of phages endow them with the capability to regulate the abundance of their hosts, thereby affecting the structure of the microbiota through cascade reactions among the bacterial communities. In our study, we expected to isolate phages of *F. nucleatum* for clinical application. Nevertheless, safety assessment of phage isolates should be the first priority for clinical use. Although phages are considered to be generally safe, as they have no effects on mammalian cells, any virulence factors and antibiotic-resistance genes they carry have the risk of being transferred to host bacteria through infection. To comprehensively understand the safety concerns, a whole genome-based safety assessment was conducted in our study. The results showed that no virulence factors or antibiotic resistance genes were predicted in the five phage genomes (Table 2). This indicates the good safety of JD-Fnp1 ~ JD-Fnp5 in medical applications. We recommend that all phages being considered for therapeutic use require a genome-wide safety assessment. A comprehensive understanding of bacteriophages at the genetic level is helpful for assessing the safety and efficacy of phages in medical applications (Cochrane et al., 2016; Kabwe et al., 2019).

*F. nucleatum* has been proven to be a high-risk factor for CRC and other diseases, and the regulation of intestinal microbiota with phages to decrease the abundance of *F. nucleatum* provides a new strategy for treatment. The isolation and characterization of five isolates of novel *F. nucleatum* phages took a solid first step for further *in vivo* studies and clinical trials. Nevertheless, the host spectrum remains a serious obstacle for application. Phage cocktails enhanced the efficacy of treating multidrug resistant bacterial infections in several studies, which may shed light on a therapeutic strategy of *F. nucleatum* phages (Dedrick et al., 2019; Leshkasheli et al., 2019; Kifelew et al., 2020; Aghaee et al., 2021; Hesse et al., 2021). It is still necessary to excavate additional phage resources and establish phage libraries that target certain crucial members of the gut microbiota.

## Data availability statement

The datasets presented in this study can be found in online repositories. The names of the repository/repositories and accession number(s) can be found below: https://www.ncbi.nlm.nih.gov/genbank/, ON464759, ON464760, ON464761, ON464762, ON464764.

## Ethics statement

The study was approved by the Ethics Committees of Shanghai Ninth People’s Hospital affiliated with Shanghai Jiao Tong University (registration number SH9H-2020-T76-2) and Renji Hospital affiliated with Shanghai Jiao Tong University (registration number 2017–205). The patients/participants provided their written informed consent to participate in this study.

## Author contributions

YW, QC, and CL contributed to conception and design of the study. XG instructed the study. YW, ZL, LY, ZX, and QC performed the experiments and recorded the experimental results. YW performed the statistical analysis. MC conducts bioinformatics related analyses. JQ, YZ, and MC contributed reagents, materials and analysis tools. YW wrote the first draft of the manuscript. GD and JH collected the human samples. All authors contributed to manuscript revision, read, and approved the submitted version.

## Funding

This work was supported by the National Natural Science Foundation of China (No. 32170141).

## Conflict of interest

The authors declare that the research was conducted in the absence of any commercial or financial relationships that could be construed as a potential conflict of interest.

## Publisher’s note

All claims expressed in this article are solely those of the authors and do not necessarily represent those of their affiliated organizations, or those of the publisher, the editors and the reviewers. Any product that may be evaluated in this article, or claim that may be made by its manufacturer, is not guaranteed or endorsed by the publisher.

## Footnotes

^1^ https://rast.nmpdr.org/

^2^ https://blast.ncbi.nlm.nih.gov/

^3^ http://gravity.cvr.gla.ac.uk

^4^ https://itol.embl.de/

^5^ https://ardb.cbcb.umd.edu/

^6^ http://www.mgc.ac.cn/VFs/main.htm

^7^ http://www.genome.jp/viptree

^8^ https://img.jgi.doe.gov/vr

^9^ https://www.ncbi.nlm.nih.gov/Structure/cdd/wrpsb.cgi

## Supplementary material

SUPPLEMENTARY FIGURE S1 *In vitro* bactericidal activity of JD-Fnp4 against *F. nucleatum* host bacteria ATCC 25586 **(A)** and ATCC 23726 **(B)**. *F. nucleatum* strain ATCC 25586 and ATCC 23726 was infected by JD-Fnp4 at MOIs of 0, 10, 100, and 1,000, respectively. The *x* axis represents the co-culture time of JD-Fnp4 phage with ATCC 25586 and ATCC 23726 respectively; the y axis represents the change of OD600 of bacteria. Data are displayed as the means ± SD (error bars) from three independent experiments.

SUPPLEMENTARY FIGURE S2 Comparison of similarity of JD-Fnp1 ~ JD-Fnp5 with FNU1, φFunu1 and metagenomics assemblies from IMG/VR v3 in the protein level with Roary (*i* = 80). Other parameters used in this analysis are -cd 99 and otherwise default parameters. Each column represents a phage’s genome or assembled phage genome from IMG/VR v3, the longitudinal direction is the proteins encoded by selected phages. The similar proteins will be combined to form a row, each row is a type of gene-encoded protein. The dark blue color represents the existence of protein. JD-Fnp1 ~ JD-Fnp5 are new *F. nucleatum* bacteriophages discovered in this study, which are shown in red. FNU1 and φFunu1 are reported *F. nucleatum* phages with whole-genome sequence data and are shown in blue. Other bacteriophages are displayed according to their serial numbers in IMG/VR v3.

SUPPLEMENTARY FIGURE S3 Conserved domains comparison of JD-Fnp1 ~ JD-Fnp5, FNU1, φFunu1, and metagenomics assemblies from IMG/VR v3. The heatmap was created with the default parameter value based on the results of conserved domain search (*e* = 0.0001), which showed the presence or absence of the CDD in the phage-related genome. The horizontal axis represents phage genomes, and the vertical axis represents conserved domains. JD-Fnp1 ~ JD-Fnp5 are new *F. nucleatum* bacteriophages discovered in this study, which are shown in red. FNU1 and φFunu1 are reported *F. nucleatum* phages with whole-genome sequence data and are shown in blue. Other bacteriophages are displayed according to their serial numbers in IMG/VR v3.
