# Supplementary material for: Isolation and characterization of novel Fusobacterium nucleatum bacteriophages
Source: Front Microbiol. 2022 Nov 3;13:945315. doi: 10.3389/fmicb.2022.945315 (PMC9670143; doi:10.3389/fmicb.2022.945315)
Supplement: SUPPLEMENTARY FIGURE S1 — In vitro bactericidal activity of JD-Fnp4 against F. nucleatum host bacteria ATCC 25586 (A) and ATCC 23726 (B). F. nucleatum strain ATCC 25586 and ATCC 23726 was infected by JD-Fnp4 at MOIs of 0, 10, 100, and 1,000, respectively. The x axis represents the co-culture time of JD-Fnp4 phage with ATCC 25586 and ATCC 23726 respectively; the y axis represents the change of OD600 of bacteria. Data are displayed as the means ± SD (error bars) from three independent experiments. [file Data_Sheet_1.zip › Table S1.DOCX]

**Table S1: General features and accession number of phages**

**used in phylogenetic tree**

| **Phage** | **Family** | **Genome size (bp)** | **Accession No.** |
| --- | --- | --- | --- |
| Fusobacterium phage FNU1 | *Siphoviridae* | 130,914 | NC_055035.1 |
| Fusobacterium phage φFunu1 | *Myoviridae* | 39,921 | KR131710.1 |
| Streptococcus phage APCM01 | *Siphoviridae* | 31,075 | NC_029030.1 |
| Streptococcus phage M102 | *Siphoviridae* | 31,147 | NC_012884.1 |
| Streptococcus phage PH10 | *Siphoviridae* | 31,276 | NC_012756.1 |
| Streptococcus phage SM1 | *Siphoviridae* | 34,692 | NC_004996.1 |
| Streptococcus phage YMC-2011 | *Siphoviridae* | 40,758 | NC_018285.1 |
| Streptococcus phage PH15 | *Siphoviridae* | 39,136 | NC_010945.1 |
| Streptococcus phage Cp-1 | *Salasmaviridae* | 19,343 | Z47794.1 |
| Streptococcus phage SOCP | *Salasmaviridae* | 19,347 | KJ617393.1 |
| Streptococcus phage Dp-1 | *Siphoviridae* | 56,506 | NC_015274.1 |
| Lactobacillus phage P1 | *Siphoviridae* | 73,787 | NC_047764.1 |
| Aggregatibacter phage S1249 | *Myoviridae* | 43,970 | NC_013597.1 |
