# Supplementary figures and images for: Isolation and characterization of novel Fusobacterium nucleatum bacteriophages
Source: Front Microbiol. 2022 Nov 3;13:945315. doi: 10.3389/fmicb.2022.945315 (PMC9670143; doi:10.3389/fmicb.2022.945315)

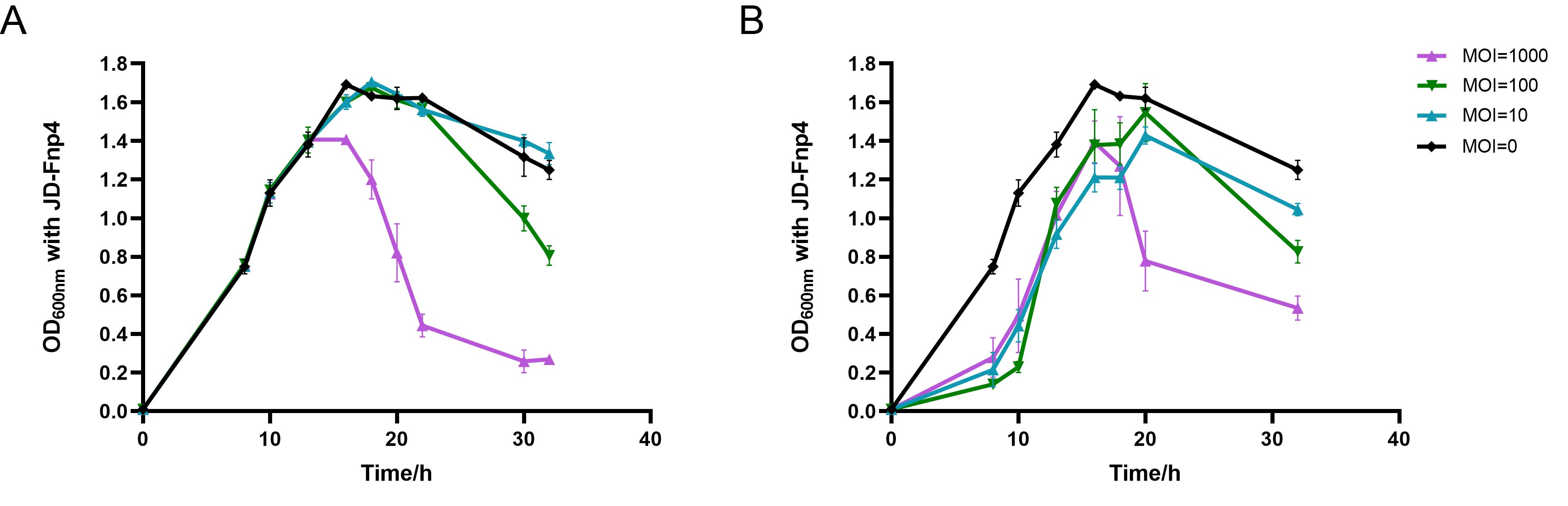

Supplement: SUPPLEMENTARY FIGURE S1 — In vitro bactericidal activity of JD-Fnp4 against F. nucleatum host bacteria ATCC 25586 (A) and ATCC 23726 (B). F. nucleatum strain ATCC 25586 and ATCC 23726 was infected by JD-Fnp4 at MOIs of 0, 10, 100, and 1,000, respectively. The x axis represents the co-culture time of JD-Fnp4 phage with ATCC 25586 and ATCC 23726 respectively; the y axis represents the change of OD600 of bacteria. Data are displayed as the means ± SD (error bars) from three independent experiments. [file Data_Sheet_1.zip › Image 1.JPEG]

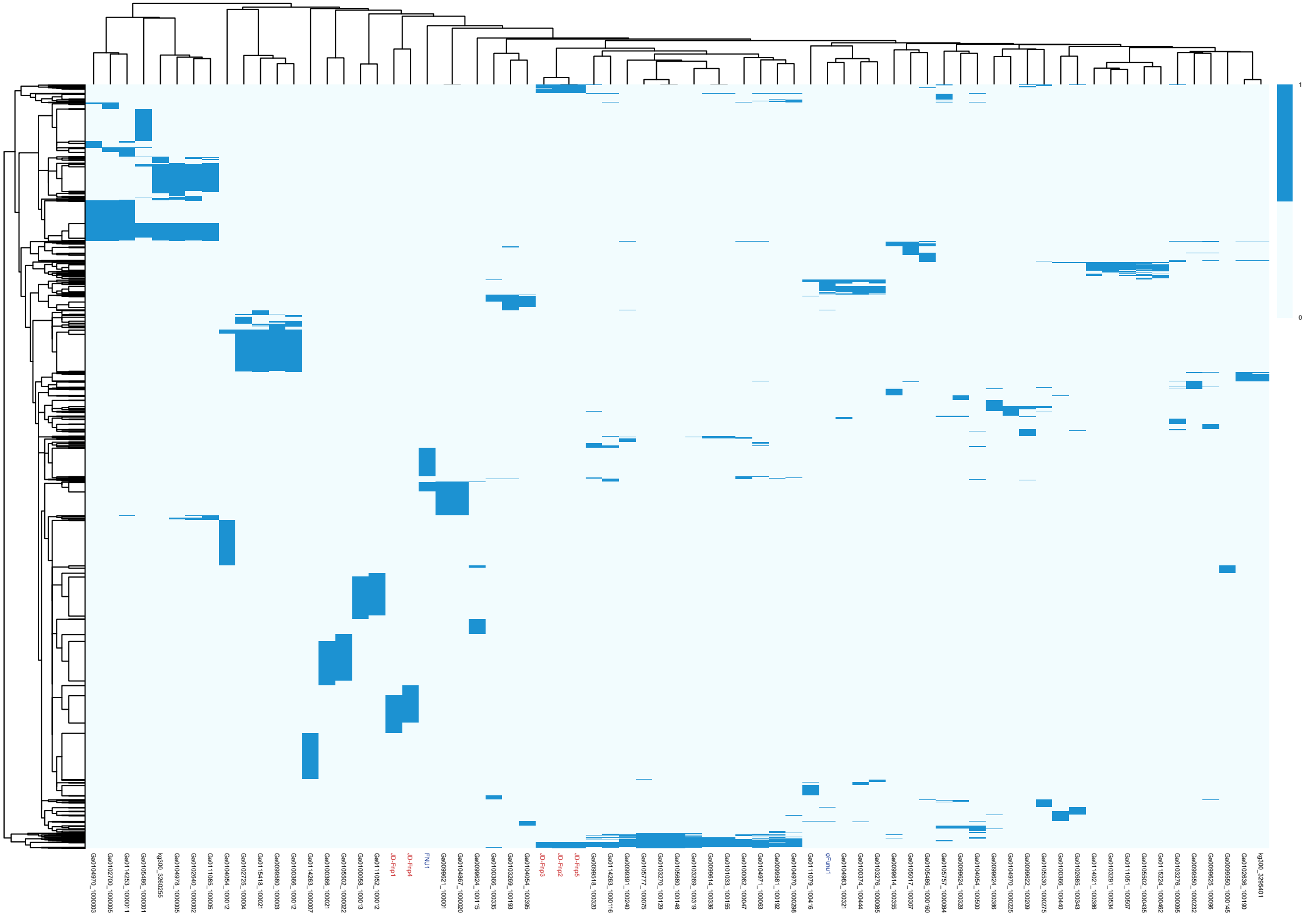

Supplement: SUPPLEMENTARY FIGURE S1 — In vitro bactericidal activity of JD-Fnp4 against F. nucleatum host bacteria ATCC 25586 (A) and ATCC 23726 (B). F. nucleatum strain ATCC 25586 and ATCC 23726 was infected by JD-Fnp4 at MOIs of 0, 10, 100, and 1,000, respectively. The x axis represents the co-culture time of JD-Fnp4 phage with ATCC 25586 and ATCC 23726 respectively; the y axis represents the change of OD600 of bacteria. Data are displayed as the means ± SD (error bars) from three independent experiments. [file Data_Sheet_1.zip › Image 2.PDF]

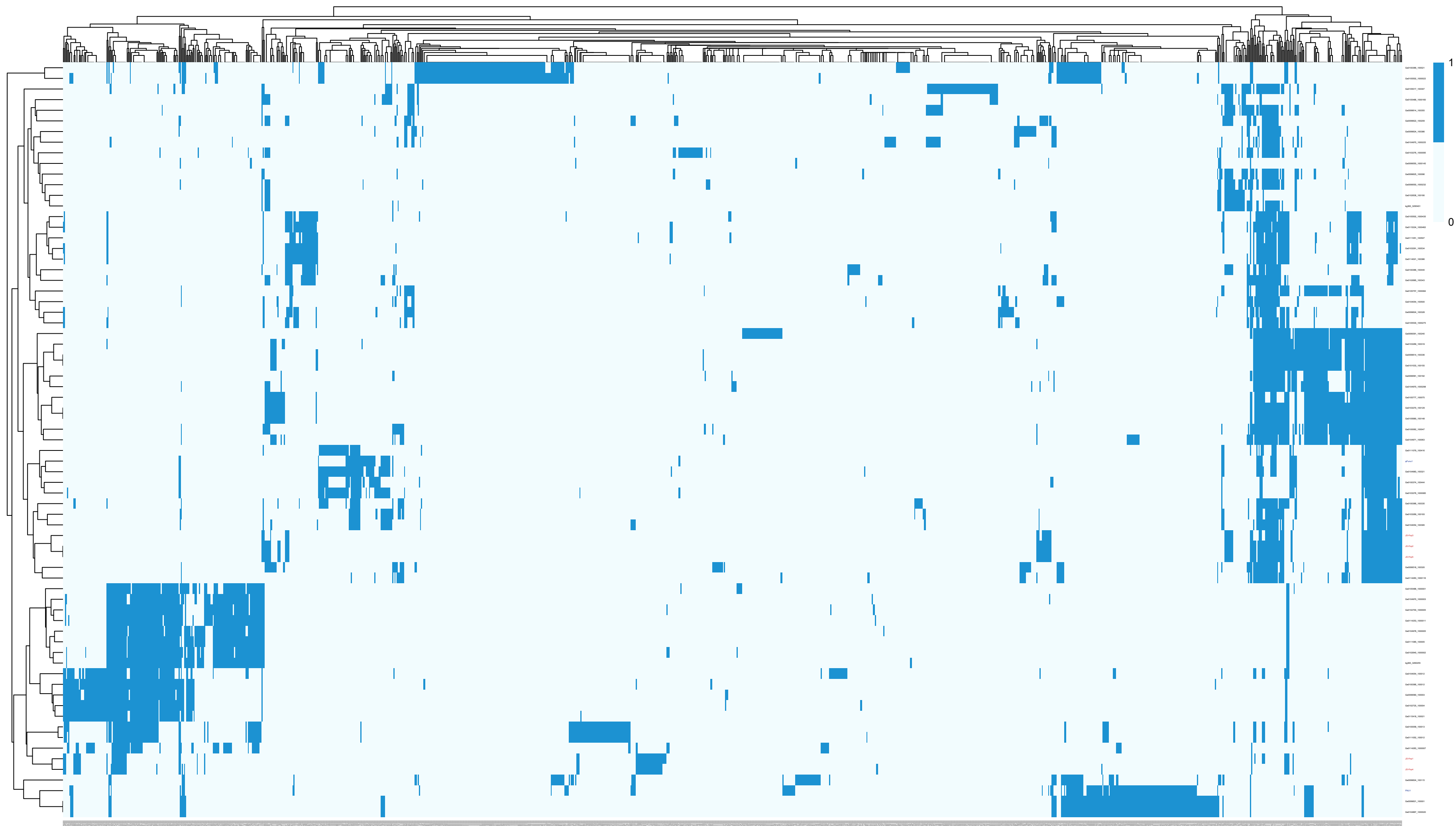

Supplement: SUPPLEMENTARY FIGURE S1 — In vitro bactericidal activity of JD-Fnp4 against F. nucleatum host bacteria ATCC 25586 (A) and ATCC 23726 (B). F. nucleatum strain ATCC 25586 and ATCC 23726 was infected by JD-Fnp4 at MOIs of 0, 10, 100, and 1,000, respectively. The x axis represents the co-culture time of JD-Fnp4 phage with ATCC 25586 and ATCC 23726 respectively; the y axis represents the change of OD600 of bacteria. Data are displayed as the means ± SD (error bars) from three independent experiments. [file Data_Sheet_1.zip › Image 3.PDF]
